# Affordable automated phenotypic antibiotic susceptibility testing method based on a contactless conductometric sensor

**DOI:** 10.1038/s41598-020-77938-7

**Published:** 2020-12-04

**Authors:** Xuzhi Zhang, Xiaoyu Jiang, Qianqian Yang, Yong Xu, Xiaochun Wang, Jinping Wang, Xiaobo Sun, Guosi Xie, Yan Zhang, Jun Zhao, Keming Qu

**Affiliations:** 1 Yellow Sea Fisheries Research Institute, Chinese Academy of Fishery Sciences, Qingdao, 266071 China; 2 Laboratory for Marine Fisheries Science and Food Production Processes, Pilot National Laboratory for Marine Science and Technology (Qingdao), Qingdao, 266071 China; 3 College of Chemical and Pharmaceutical Sciences, Qingdao Agricultural University, Qingdao, 266109 China

**Keywords:** Biological techniques, Biotechnology, Microbiology

## Abstract

User-friendly phenotypic antibiotic susceptibility testing (AST) methods are urgently needed in many fields including clinical medicine, epidemiological studies and drug research. Herein, we report a convenient and cost-effective phenotypic AST method based on online monitoring bacterial growth with a developed 8-channel contactless conductometric sensor (CCS). Using *E. coli* and *V. parahaemolyticus* as microorganism models, as well as enoxacin, florfenicol, ampicillin, kanamycin and sulfadiazine as antibiotic probes. The minimum inhibitory concentration (MIC) determination was validated in comparison with standard broth microdilution (BMD) assay. The total essential agreements between the CCS AST assays and the reference BMD AST assays are 68.8–92.3%. The CCS has an approximate price of $9,000 (USD). Requiring neither chemical nor biotic auxiliary materials for the assay makes the cost of each sample < $1. The MICs obtained with the automated CCS AST assays are more precise than those obtained with the manual BMD. Moreover, in 72 percent of the counterpart, the MICs obtained with the CCS AST assays are higher than that obtained with the BMD AST assays. The proposed CCS AST method has advantages in affordability, accuracy, sensitivity and user-friendliness.

## Introduction

Determining the susceptibility of microorganisms towards target agents (e.g., antibiotics and nanoparticles) is of great significance in many fields including medicine, epidemiology, drug research, environmental surveys and agriculture^1–3^. Antimicrobial susceptibility testing (AST) is the concentration of antimicrobial agents that is required to inhibit proliferation of target microorganisms in vitro and is the most valuable tool for determination^1,4^. To achieve rapid, reproducible and accurate measurements, automated AST methods have been developed in recent decades, and most available methods are divided into two categories based on mechanism: genotypic and phenotypic methods.

Genotypic AST methods attempt to determine specific resistance genes or genetic mutations using molecular amplification^4^ or sequencing^5^ approaches. In most of these performances, the requirements of microbial growth are bypassed resulting in a fast response; the results can be obtained with high specificity and sensitivity within 2 h^4,6^. An intrinsic limitation of all genotypic methods is that they detect only the potential for resistance. Thus, the susceptibility has to be validated with phenotypic tests^1,7^. Moreover, the other drawback with genotypic methods is that only known sequences associated with resistance can be targeted. Not only are there many more sequences that have yet to be elucidated, but new forms of resistance are out of reach^2^. In contrast, phenotypic AST methods analyze the effect of target agents on characteristics of microbial growth. They can provide qualitative data for the strain tested as well as quantitative values of the minimum inhibitory concentration (MIC, i.e., the lowest antibiotic concentration that inhibits visible growth)^4,7–10^. To date, phenotypic AST methods are still considered the gold standard and are the most popular because of their universal detection of resistance irrespective of the resistance mechanisms^2,8^.

To improve the efficiency of phenotypic AST assays, various semi-automatic and automatic readout approaches have been introduced as reviewed previously^1,4,11–13^. In general, optics^2,9,12–16^, electrochemistry^12,17–20^, microcalorimetry^21^, mass sensor^22^, mass spectrometry^23^ and gene analysis^8^ techniques are used for the readout of microbial growth. These developments offer fast susceptibility results making measurements simple versus manual plate counting. Though most of these AST methods are still in the proof-of-concept status, those based on on-line monitoring microbial growth with optical devices have been implemented widely to conduct phenotypic assays^15,16^, because they also offer accuracy and reliability, as well as the capacity for quantitative MIC results in a non-destructive and high-throughput manner.

Several commercial systems, including MicroScan WalkAway, Vitek, BD Phoenix and Sensititre, are already moving into clinical spaces^13,24^. These inventions can speed up AST, increase consistency of susceptibility results across different locations, and reduce the burden of work for users. For example, the susceptibility profile of up to 96 microbial samples can be determined using the automated broth microdilution (BMD) assay through on-line optical density (OD) measurements within 20 h^25^_,_ despite there being no official standardized protocol for OD measurements. Automatic readout approaches can lower the accuracy depending on the skill of operators. However, the cost stunts their widespread applications, especially in the developing world^4,5,26^. They might also be unreliable when target microorganisms are cultured in the presence of substrates that may interfere with the optical signal or only proliferate when attached to base material surfaces^4^.

Electrochemical readout requires only simple electronics for direct electronic detection of microbial growth. This can bypass the requirements of complex optical-electric conversion^18^. Thus, instruments are easier to miniaturize and become more cost-effective versus optical systems. Traditionally, the working electrode is galvanic contact with liquid broth or solution. This invasive manner inevitably causes environmental perturbations on microbial growth as well as undesirable electrode deterioration and non-specific fouling^27^ that can undermine the accuracy of on-line monitoring^12,19^. Therefore, an electrochemical readout approach that can be used to monitor microbial growth in real time with non-invasive manner is urgently needed for developing practical AST methods.

To meet this goal, we have constructed a contactless conductometric sensor (CCS) based on a multi-channel capacitively-coupled contactless conductivity detector (C^4^D)^28^. The measurement based on this instrument offers several advantages over classical electrochemical- and turbidity/absorbance-based approaches. For instance, it is superior to optical-based methods in that turbidity and other optical interferences are significant issues^29^. Versus impedance sensors, this method exhibits better reproducibility and accuracy with high temporal resolution^17,30^. Moreover, unlike other electrochemical methods, this system requires neither chemical, biotic, or physical compounds as indicators or auxiliary materials, nor any immobilizing steps. This effectively reduces the cost and complexity for on-line monitoring of microbial growth^12^.

Here, we report a novel AST method by modifying a common BMD assay. An 8-channel CCS, which allows simultaneous cultivation and on-line analysis of growth inhibition, is developed and characterized. Taking *E. coli* and *V. parahaemolyticus* as microorganism model and enoxacin, florfenicol, ampicillin, kanamycin and sulfadiazine as antibiotic probes, we validated the capability of the new method for achieving MIC determination. Our goal is to overcome the limited applicability of existing methods for studying the dynamic effects of antibiotics on microbial growth kinetics, and to provide an affordable and simple tool for phenotypic AST assays.

## Materials and methods

### Materials and reagents

Standard bacterial strains of *E. coli* (ATCC35150) and *V. parahaemolyticus* (ATCC17802) were purchased from BIOBW Biotechnology Co., Ltd (Beijing, China). *E. coli* and *V. parahaemolyticus* isolates were obtained from fishery water and the shrimp *Penaeus vannamei*, respectively. Enoxacin, florfenicol, ampicillin, kanamycin and sulfadiazine were purchased from Sigma-Aldrich (St. Louis, MO, USA). Other common chemicals were purchased from the Shanghai Chemical Reagent Co. (Shanghai, China) and were of analytical grade. Unless otherwise indicated, liquid broths and solutions were prepared with ultrapure water (resistivity: 18.2 MΩ cm at 25 °C) from a Master Touch-RUV water purification system (Hitech Instruments Co., Ltd., Shanghai, China).

*Escherichia coli* was aerobically cultured in liquid Luria–Bertani (LB) broth. *V. parahaemolyticus* was aerobically cultured in liquid 2216E broth (a common complex culture broth for marine bacteria, consisting of 0.5% tryptone, 0.1% yeast extract, 3.4% NaCl and 0.01% FePO_4_, pH 7.6–7.8). Both of the broths were purchased from the Hope Bio-Technology Co., Ltd (Qingdao, China). Bacterial cultivation was conducted in accordance with previously described methods^28,31^ with minor modifications. Briefly, strains were stored at − 80 °C and then pre-grown overnight in the appropriate broth with constant shaking. Unless otherwise indicated, the incubation temperature for *E. coli* and *V. parahaemolyticus* was 37 °C and 28 °C, respectively. Active strains were then further transferred to new culture broth. After a second incubation for ~ 10 h, the cell numbers were measured with an OD method according to Clinical and Laboratory Standards Institute (CLSI) guidelines^32^. The results were then validated with a plate-counting method used previously^28^. The cultures were immediately diluted to achieve a cell concentration of 10^9^ CFU/mL for further use.

### Fabrication and characterization of the CCS

The 8-channel CCS was developed on the basis of one prototype we constructed previously^28^. In brief, a miniature electronic fan (MA1062, Sunon Technology Development Co., Ltd, Beijing, China), a programmable temperature sensor (WH801, Wattion Electronic Control System Co., Ltd, Guangzhou, China) and a thermoelectric cooler (TEC1-12706, Changshengyongxing Co., Ltd, Shenzhen, China) were used to guarantee expected identical temperature inside the working chamber of the sensor. A developed eight-channel C^4^D (manufactured by eDAQ Pty Ltd., Sydney, Australia), including the software TERA TERM, was used to monitor the conductivity changes of the liquid broth on-line in test tubes. Note, in order to obtain stable and sensitive conductivity change values for the liquid broth over the range of 15–45 mS/cm, the geometric parameters of the working electrodes were developed according to Equations 1–5 from the literature^33^. Here, in each channel a couple of copper cylinders (ID = 5.01 mm; thickness = 0.5 mm; length = 2.0 mm) were used as the actuator electrode and pick-up electrode. The distance between the actuator electrode and pick-up electrode was 8.0 mm. The excitation frequency and excitation amplitude were 500 K Hz and 16 V, respectively. The collection period of apparent conductivity value can be selected over a range of 1/100 s to 20 min^28,34^. The characteristics of the electronic sensor, including the uniformity of temperature in the working chamber and the robustness and reproducibility of the C^4^D system, were characterized with the same methods as used previously^28^ (see Supplementary Information).

### Characterizing growth behaviors of *E. coli* and *V. parahaemolyticus* with the CCS

To characterize the growth behavior of viable *E. coli*, bacterial cells at desired concentrations in liquid LB broth (2 mL in total, containing 0.5% NaCl) were loaded into test tubes (NORELL tubes, OD = 5.0 mm, ID = 4.2 mm, length = 203.0 mm, volume ≈ 2.8 mL, Norell, Inc., Morganton, USA). The tube openings were then covered with gas-permeable films. Meanwhile, a control sample without inoculation was prepared similarly and loaded into another test tube. Each tube was then inserted into a separate channel of the CCS in which the temperature was 37 °C. After an incubation of 120 s (to balance the temperature inside and outside of the tubes)^28^, apparent conductivity values were collected every 30 s. The apparent conductivity data from each tube were blanked by subtracting the first recordings from the remaining values to form normalized apparent conductivity values (NACVs, showed in voltage). The CCS growth curves were then generated by plotting NACVs as a function of incubation times. In the case of characterizing growth behavior of viable* V. parahaemolyticus* at 28 °C, liquid 2216E broth was used instead of LB broth.

### CCS AST assay

**Scheme 1:**
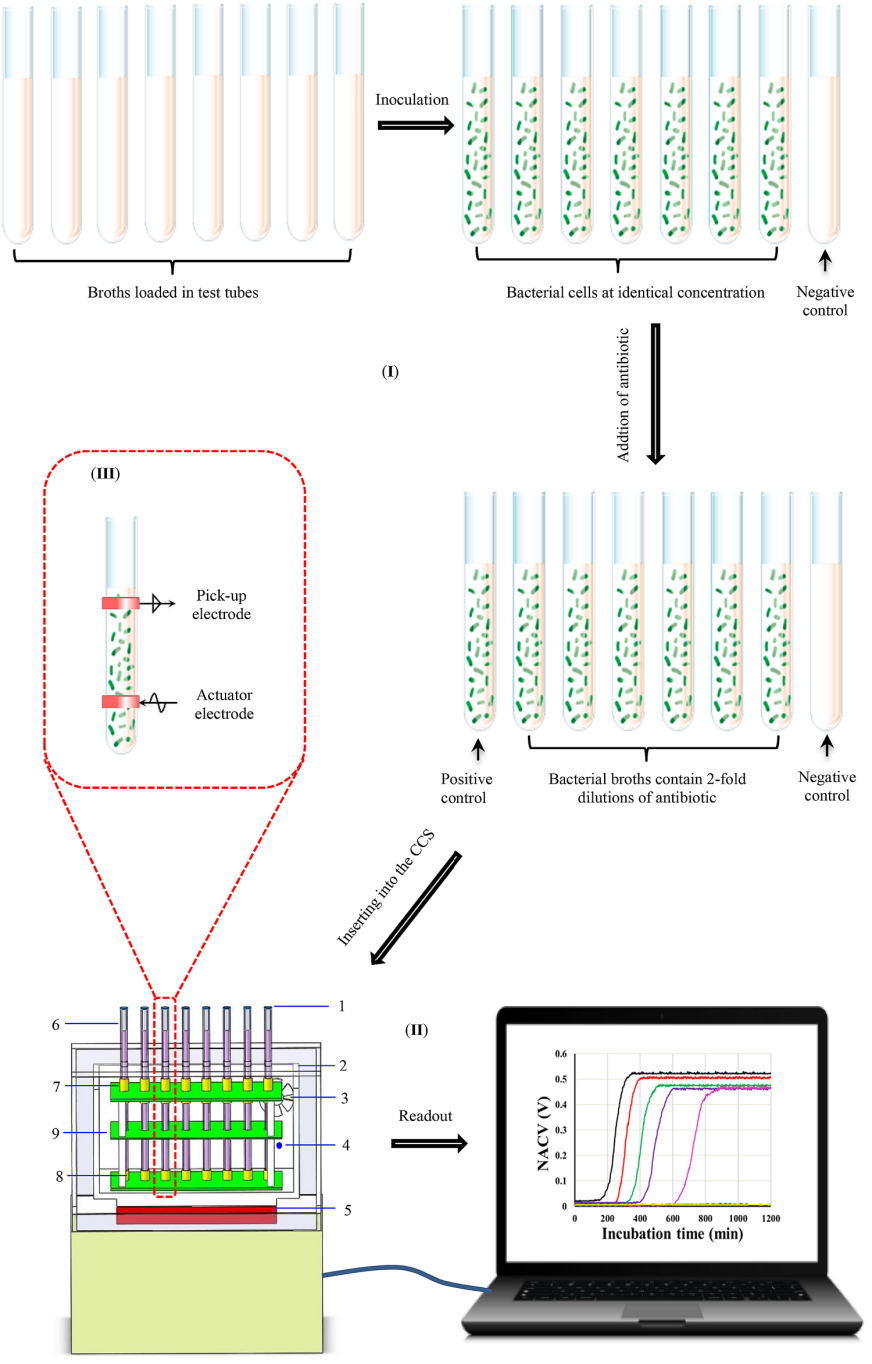
Schematic diagram of the workflow of phenotypic AST assay with the 8-channel CCS. (I) Preparation of AST samples; (II) Incubation and automated generation of growth curves with the CCS controlled by a computer. 1—gas-permeable film; 2—thermal insulator; 3—mini electronic fan; 4—temperature sensor; 5—thermoelectric cooler; 6—test tube; 7—actuator electrode; 8—pick-up electrode; and 9—grounded Faraday shield to avoid direct capacitive coupling between the actuator electrode and pick-up electrode^33^. (III) The illustration of the couple of working electrodes outside the test tube.

Scheme 1 shows that the procedure of the phenotypic AST assay with the CCS consisted of two steps: Preparation of AST samples and incubation/read out. Re-suspended bacterial cells in liquid broth were added to each tube for a final concentration of approximately 5 × 10^5^ CFU/mL^23^. In the test tubes, serial twofold dilutions of antibiotics were made in liquid broth. After covering the tube opening with a gas-permeable film, we simultaneously inserted all tubes into a separate channel of the CCS and collected the NACVs as stated in “Characterizing growth behaviors of *E. coli* and *V. parahaemolyticus* with the CCS” for 20 h. The liquid broth with and without bacterial cells were taken as positive and negative controls, respectively. In total, 2400 datapoints were obtained for every tested sample. Bacterial growth curves were simultaneously generated by plotting NACVs against incubation times. The MIC for each antibiotic is defined as the lowest antibiotic concentration, which inhibits the growth of the target microorganism, as assessed from the absence of sigmoidal curve^9^.

### BMD AST assay

In the reference experiments, the MICs of these five kinds of antibiotics against *E. coli* and *V. parahaemolyticus* were determined by the BMD method according to the CLSI guidelines^32^. Briefly, each antibiotic was measured in two-fold concentrations over a desired range in Corning 96-well plates (Corning Incorporated, USA). The final bacterial inoculum in the measurement was approximately 5 × 10^5^ CFU/mL. In each well, 100 μL LB or 2216E was used as nutrient broth for *E. coli* and *V. parahaemolyticus*, respectively. Plates were incubated for 20 h. The MIC was defined as the lowest concentration of antibiotic, resulting in complete inhibition of growth as determined visually^23^.

### Precision and validity analysis

A precision analysis was conducted referring to Kenneth and Kirby’s report^35^ with minor modifications. Briefly, CCS AST and BMD AST assays were repeated 11 times in triplicate for each antibiotic-microorganism combination using kanamycin as an antibiotic probe. All performances occurred on separate days with freshly prepared antibiotic dilutions and independent inocula were used to determine the MICs. The distributions of determined MICs from the two assays were analyzed in comparison. Validity analysis was conducted with essential agreement (EA) levels using 65 *E. coli* isolates and 16 V*. parahaemolyticus* isolates against enoxacin, florfenicol, ampicillin, kanamycin and sulfadiazine. Each MIC value determined by the CCS AST assay was compared with the counterpart determined by the BMD AST assay. The CCS AST assay was considered to have an evaluable EA if its MIC was within ± 200% of that obtained with the reference assay. In addition, when the proportion of MIC obtained with the CCS AST assay to that obtained with the reference assay was >  ± 2 but ≤  ± 3, it was calculated as the minor error (mE).

## Results

### Characteristics of the CCS

Bacterial growth is temperature-sensitive as is the response of C^4^D^36^. Thus, the temperature control features were characterized first. Figure S1 (Supporting information) shows the curves of 50.0 and 120.0 mM KCl solutions simultaneously measured in separate channels. As expected, the variation in apparent conductivity was very small (< 0.8%) over a period of 20 h in both cases. These results demonstrate the uniformity of temperature in the working chamber as well as robust and identical conductometric measurements. The difference of apparent conductivity between separate channels from the same concentration of KCl solution resulted from minor variations in the geometry size of the tubes and their coupling to the electrodes^28^. For monitoring bacterial growth, the critical factor of measurement was to record conductivity changes rather than absolute conductivity values. Thus, these differences between separate channels have no influence on forming bacterial growth curves because of our normalized algorithm. There is a linear relationship between the concentration of KCl solutions and apparent conductivity values with a slope of 562 ± 3 mV per mS/cm (*R*^2^ ≥ 0.9910) from 20.0 to 200.0 mM, thus suggesting a high sensitivity.

### Growth behaviors of *E. coli* and *V. parahaemolyticus*

**Figure 1:**
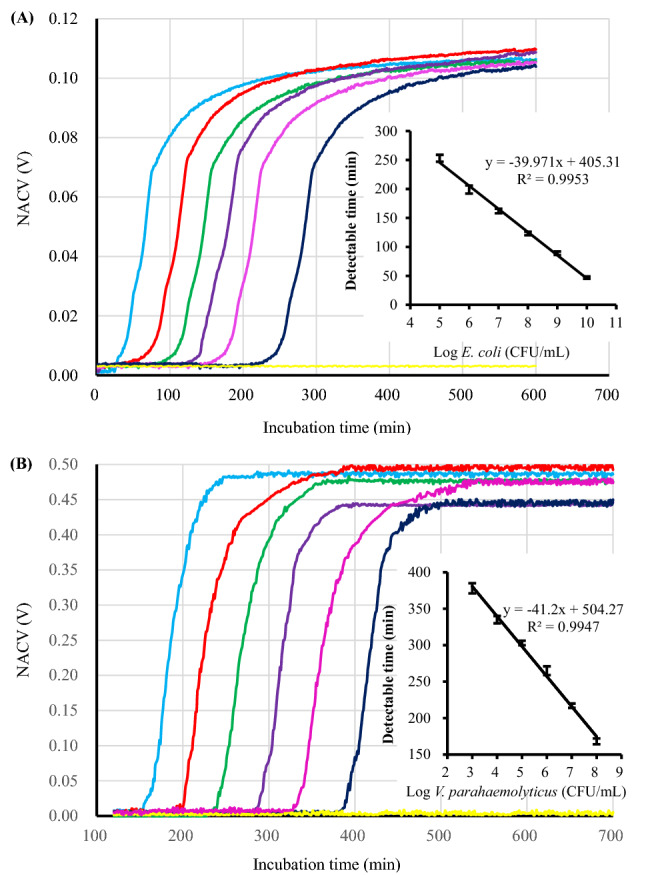
CCS growth curves (NACV *vs*. incubation time) of *E. coli* in liquid LB broth (**A**) and *V. parahaemolyticus* in liquid 2216E broth (**B**). (**A**): from left to right, the initial inoculum of *E. coli* was 10^10^, 10^9^, 10^8^, 10^7^, 10^6^ and 10^5^ CFU, respectively. Insert: The linear relationship between the logarithm of initial *E. coli* and detectable time. (**B**): from left to right, the initial inoculum of *V. parahaemolyticus* was 10^8^, 10^7^, 10^6^, 10^5^, 10^4^ and 10^3^ CFU, respectively. Insert: The linear relationship between the logarithm of initial *V. parahaemolyticus* and detectable time. Yellow horizontal lines show the results of negative control experiments. NACV values of liquid broths in each test tube were collected at an interval of 30 s with excitation frequency of 500 K Hz and excitation amplitude of 16 V.

The conductivities of culture broths in six test tubes, in which the initial concentration of *E. coli* cells were 10^3^, 10^4^, 10^5^, 10^6^, 10^7^ and 10^8^ CFU/mL, respectively, were monitored simultaneously with the CCS. As shown in Fig. 1A, six sigmoidal CCS growth curves presents, meaning that the system can characterize the growth rate of viable bacteria as well as estimate the initial concentration of the initial bacterial suspension^28,37^. For simplicity, the time needed for the NACV of the broth to reach 0.02 V is defined as a “detectable time.” There is a linear relationship between the logarithmic values of initial inoculum of *E. coli* and detectable times over the tested range with a correlation coefficient (*R*^2^) of 0.9953 (Fig. 1A insert). For the negative control (in yellow), no sigmoidal growth curve was observed over the same time span, suggesting that without viable bacteria there is no conductivity change during the incubation. These outcomes are in excellent agreement with those observed with an earlier generation prototype^28^.

Using the previous prototype^28^, we can obtain stable and sensitive conductivity change values for the liquid broth over the range of 9–14 mS/cm. With the latest version, we can obtain stable and sensitive conductivity change values for the liquid broth over the range of 15–45 mS/cm. This wider working window makes it possible to monitor bacterial growth in common liquid broths, e.g., LB and 2216E.

The conductivities of culture broths in five test tubes, in which the initial concentration of *E. coli* cells were all 10^3^ CFU/mL, were monitored simultaneously with the CCS. The standard deviation of detectable times is 3.2 min, indicating a good accuracy.

For *V. parahaemolyticus*, the growth of serial inocula at concentrations of 10^3^, 10^4^, 10^5^, 10^6^, 10^7^ and 10^8^ CFU/mL in liquid 2216E broth was simultaneously monitored with the CCS. As shown in Fig. 1B, six sigmoidal growth curves are observed. Similar to *E. coli*, the duration of the lag phase is proportional to the initial inoculum of bacteria. There is a linear relationship between the logarithmic values of initial inoculum of *V. parahaemolyticus* and detectable times over the tested range with a correlation coefficient (*R*^2^) of 0.9947 (Fig. 1B insert). No sigmoidal growth curve was observed during the incubation when the initial inoculum was zero (negative control).

### CCS AST assay

**Figure 2:**
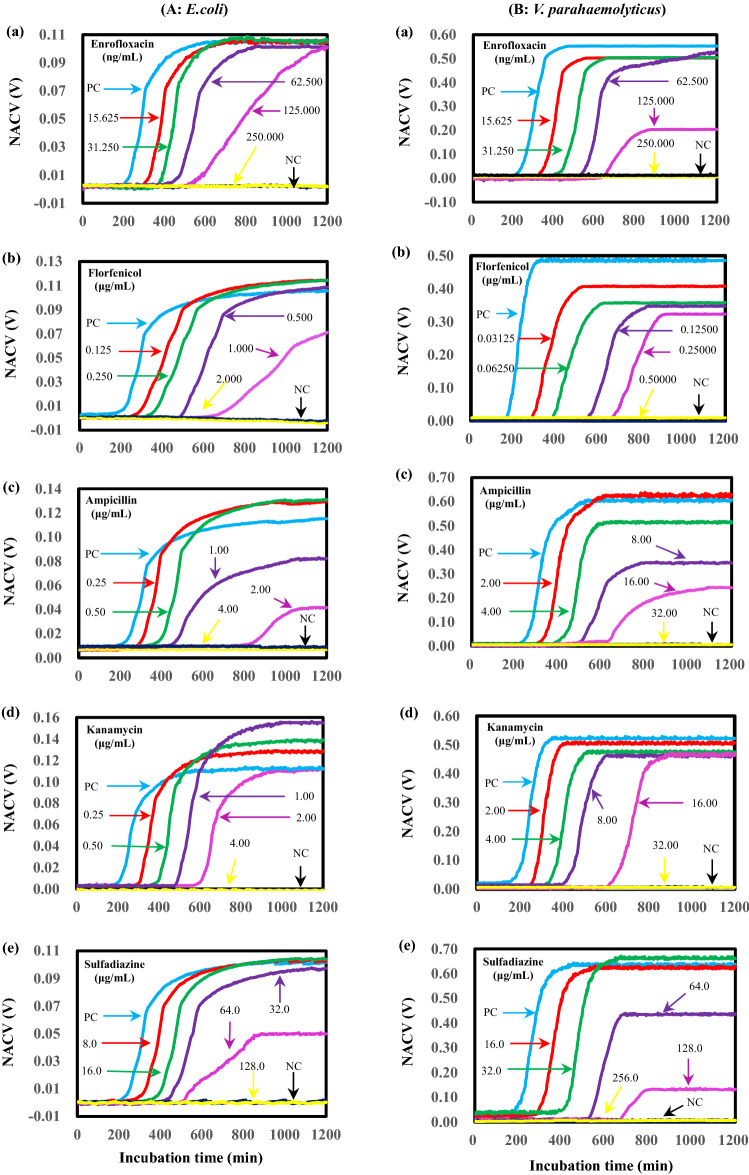
CCS growth curves (NACV *vs*. incubation time) of *E. coli* (**A**) and *V. parahaemolyticus* (**B**) in the presence of antibiotics. *E. coli* in liquid LB broth and *V. parahaemolyticus* in liquid 2216E broth were aerobically incubated at 37 °C and 28 °C, respectively.

*E.coli* ATCC 35150 in liquid LB broth was cultured in the presence of enoxacin, florfenicol, ampicillin, kanamycin, or sulfadiazine. Figure 2A shows CCS growth curves. For the positive control (with bacteria but without antibiotic) samples, sigmoidal curves were obtained as expected. We note that these curves have considerably higher temporal resolution (at an interval of 0.5 min) than those obtained with the OD method^26^ and the electrochemical method^17^. There is a lag phase for approximately 210 min; this is possibly caused by the stress that bacteria might experience after dilution and/or a loading step as well as the time required for generating enough end products to produce detectable increasing conductivity^28^. The lag phase is followed by an acceleration phase during which the growth rate increases until a constant growth rate is achieved, i.e., entering the exponential phase. Subsequently, the growth rate begins to decline to form a deceleration phase. Compared to the positive control, the lag phase duration of bacteria regularly extends with increasing antibiotic concentration, thereby contributing to a delayed onset of growth when microorganisms are exposed to sub-lethal antibiotic concentrations. These data suggest that all five antibiotics show concentration-dependent effects on *E. coli* growth dynamics^38^.

Similar maximum growth rates and maximum growths were found in the presence of 15.625, 31.250 and 62.500 ng/mL enoxacin despite the onset of growth delays with the increase of antibiotic concentration (Fig. 2A(a)). In contrast, the maximum growth rate of *E. coli* is lower in the presence of 125.000 ng/mL enoxacin, though the final NACV at the measurement endpoint can reach a similar value to the positive control. In the presence of 0.250 μg/mL enoxacin, no sigmoidal growth curve was observed over the same incubation, similar to the response of the negative control. This complete inhibition suggests an MIC of 0.250 μg/mL. The presence of low concentrations of florfenicol (0.125, 0.250 and 0.500 μg/mL) delayed the onset of *E. coli* growth and slightly lowered the maximum growth rate. The presence of 1.000 μg/mL significantly impacted the maximum growth rate as well as the maximum growth (Fig. 2A(b)). Interestingly, though the presence of 0.25 and 0.50 μg/mL ampicillin delayed the onset of growth, it did not impact the maximum growth rate. Meanwhile, the final NACV values are higher than that of the positive control, indicating that growth stimulation might have occurred^39^. However, the maximum growth rate and maximum growth were both depressed by 1.00 and 2.00 μg/mL ampicillin (Fig. 2A(c)).

Figure 2A(d) shows the response of kanamycin against *E. coli*. The maximum growth increases regularly with increases in antibiotic concentration from 0.25–1.00 μg/mL with an identical maximum growth rate similar to that in the positive control. Even in the presence of 2.00 μg/mL kanamycin, the maximum growth rate is similar to that of the positive control; the maximum growth at the endpoint of each measurement can reach a similar level for the positive control. The impact of sulfadiazine against *E. coli* is similar to that of florfenicol: the 8.0, 16.0 and 32.0 μg/mL antibiotics have little impact on the maximum growth rate and maximum growth. The presence of 64.0 μg/mL florfenicol depresses both the maximum growth rate and maximum growth (Fig. 2A(e)). These phenomena confirm that—in contrast to the duration of the lag phase—some antibiotics have neither a maximum specific growth rate nor a maximum growth (final cell amount). Thus, these latter two metrics are not reliable predictors for indicating a concentration-dependent inhibitory effect^38^. Microbial growth is completely inhibited when the concentration of the antibiotic is equal to or higher than the MIC. Thus, in the presence of high concentrations of antibiotics (value ≥ MIC), the responses of cultures are the same as that obtained with a negative controls ( see the NC lines in Fig. 2A).

Using the CCS, we obtained similar responses of enoxacin, florfenicol, ampicillin, kanamycin and sulfadiazine against *V. parahaemolyticus* (ATCC17802) growth in liquid 2216E broth. Lag phase durations of bacteria extend with increases in antibiotic concentration, suggesting that they all show concentration-dependent effects on growth dynamics. The sigmoidal CCS curves for the positive control samples (with bacteria but without antibiotic) have lag phases of ~ 220 min (Fig. 2B). In the presence of 15.625, 31.250 and 62.500 ng/mL enoxacin, despite the onset of growth delays with an increase in enoxacin concentration, there are similar maximum growth rates and maximum growths. The maximum growth rate and maximum growth are both obviously depressed in the presence of 125.000 ng/mL antibiotic. In the presence of 0.250 μg/mL enoxacin, no sigmoidal growth curve is observed over the same time span like the response of the negative control (Fig. 2B(a)). The onset of *V. parahaemolyticus* growth is also delayed by florfenicol. Unlike the case of enoxacin, the increase of florfenicol concentration from 0.03125 to 0.12500 μg/mL regularly depresses both the maximum growth rate and the maximum growth; 0.500 μg/mL florfenicol can completely inhibit the growth (Fig. 2B(b)). Interestingly, although the presence of 2.00 μg/mL ampicillin delays the onset of growth, it does not impact the maximum growth rate or maximum growth. However, the maximum growth rate and maximum growth both increasingly decreased with increasing ampicillin concentration (4.00–16.00 μg/mL) (Fig. 2B(c)). Figure 2B(d) shows the typical response of kanamycin against *V. parahaemolyticus*. Over the range of 2.00–16.00 μg/mL, the maximum growth rate and maximum growth are only slightly impacted by the presence of kanamycin. Figure 2B(e) shows the typical response of sulfadiazine against *V. parahaemolyticus*. The antibiotic at a concentration of 16.0 and 32.0 μg/mL invisibly impacts the maximum growth rate and maximum growth. The maximum growth rate and maximum growth are both increasingly depressed by the increase of ampicillin concentration from 64.00 to 128.00 μg/mL.

### BMD AST assay

The responses of *E. coli* and *V. parahaemolyticus* to enoxacin, florfenicol, ampicillin, kanamycin and sulfadiazine were characterized with BMD AST assays (Typical resulting pictures are shown in Figure S2). The resulting MICs are listed in Table 1 in comparison with those obtained with the CCS AST assays. The MICs of enoxacin against *E. coli* obtained with either BMD AST assay or CCS AST assay are much lower than that documented in the CLSI (Revised-2014)^39^. The MICs of kanamycin against *E. coli* obtained with BMD AST assay and CCS AST assay are also lower than that documented in the CLSI (Revised-2014)^39^. However, the MIC of ampicillin against *V. parahaemolyticus* obtained with CCS AST agrees well with that reported by Lopatek et al.^40^.

**Table 1:** Results of BMD AST assay in comparison with that of CCS AST assay.

Antibiotics	Bacterial species	CCS MICs (μg/mL)	BMD MICs (μg/mL)	Reference MICs (μg/mL)
Enoxacin	*E. coli*	0. 25	0. 25	2–8^39^
Florfenicol	*E. coli*	2.00	1.00	–
Ampicillin	*E. coli*	4.00	1.00	–
Kanamycin	*E. coli*	4.00	2.00	16–64^39^
Sulfadiazine	*E. coli*	128.0	64.0	–
Enoxacin	*V. parahaemolyticus*	0.250	0.125	–
Florfenicol	*V. parahaemolyticus*	0.500	0.250	–
Ampicillin	*V. parahaemolyticus*	32.00	8.00	32^40^
Kanamycin	*V. parahaemolyticus*	32.00	16.00	–
Sulfadiazine	*V. parahaemolyticus*	256.0	128.0	–

### Precision and validity

In the precision analysis, 87.9% (29/33) *E. coli* ATCC 35,150 samples show MIC of kanamycin is 4.00 μg/mL obtained with the CCS AST assay. In contrast, 78.8% (26/33) *E. coli* ATCC 35,150 samples show a MIC of 2.00 μg/mL with the BMD AST assay. In the case of *V. parahaemolyticus* (ATCC17802), 84.8% (28/33) of the samples show that the MIC of kanamycin is 32.00 μg/mL obtained with the CCS AST assay. The BMD assay offers 72.7% (24/33) samples with a MIC of 16.00 μg/mL. These findings show that the CCS AST assay is superior to the BMD AST assay in terms of precision.

In total, 81 isolated bacterial strains, including 65 *E. coli* and 16 V*. parahaemolyticus*, were used to validate the CCS AST method for the five antibiotics, also using BMD AST method as reference. The results are similar to those obtained with standard strains (Table 2). With the evaluation parameters suggested by the FDA^9^, we calculated the EA, as well as discrepancies defined as mE, by comparing the MICs of the same isolate determined with CCS AST assay and that with BMD AST assay.

**Table 2:** Validity analysis of the CCS AST assay in comparison with the BMD AST assay.

Antibiotics	Bacterial species	Total isolates	CCS MICs (μg/mL)	BMD MICs (μg/mL)	EA%	mE%
Enoxacin	*E. coli*	65	0.125–8.000	0.125–4.000	86.5	10.4
Florfenicol	*E. coli*	65	1.00–64.00	0.50–64.00	76.9	23.1
Ampicillin	*E. coli*	65	1.00–64.00	0.500–32.00	84.6	13.8
Kanamycin	*E. coli*	65	2.00–128.00	1.00–128.00	92.3	7.7
Sulfadiazine	*E. coli*	65	64.00–512.00	32.00–256.00	89.2	10.8
Enoxacin	*V. parahaemolyticus*	16	0.125–16.000	0.125–16.000	68.8	31.2
Florfenicol	*V. parahaemolyticus*	16	0.25–64.00	0.25–64.00	87.5	6.3
Ampicillin	*V. parahaemolyticus*	16	16.00–128.00	8.00–64.00	81.3	12.5
Kanamycin	*V. parahaemolyticus*	16	16.00–128.00	8.00–64.00	87.5	12.5
Sulfadiazine	*V. parahaemolyticus*	16	128.0–1024.0	64.0–1024.0	75.0	25.0

## Discussion

MIC measurements are important in medicine, epidemiology, drug research, environmental surveys, agricultural production^1–3^ and materials research^38^. Liquid suspension growth-based methods, such as the BMD, are gold standards for phenotypic AST assays. To monitor growth with real-time patterns, AST assays can yield results sooner than solid-phase tests^2^. Traditional manual BMD assays tend to be very laborious with poor precision; thus, various semi-automatic and automatic methods have been developed in the past decades^1,4^. These methods include on-line monitoring of microbial growth with OD patterns, which have good practicability and have been successful commercially because of their accuracy, reliability and non-destructive and high-throughput nature^13^. However, there are still two salient issues: (1) the optical interference from complex substrates in the broth and from adherent cells in proliferation^37^; and (2) the price of the instrument and cost of each sample test^4^. These challenges provided the motivation to develop the CCS AST method proposed herein.

The growth of bacteria transforms uncharged or weakly charged substrates, e.g., yeast, peptone and sugar into highly charged end products, such as amino acids, aldehydes, ketones, acids and other metabolites, causing a conductivity increase of the liquid broth^41^. This change can be monitored on-line with the sensitive CCS to generate bacterial growth curves by plotting NACVs as a function of incubation time. Using this approach, we could monitor up to eight samples for high-throughput testing.

Using standard bacterial strains of *E. coli* (ATCC35150) and *V. parahaemolyticus* (ATCC17802) as model microorganisms, the total EAs and mEs between the CCS AST assays and the reference BMD AST assays were found to be 80% and 20%, respectively. For *E. coli* isolates, the EAs of enoxacin, florfenicol, ampicillin, kanamycin and sulfadiazine were no lower than 76.9%. Meanwhile, the mEs were 7.7–23.1%. For *V. parahaemolyticus* isolates, the EAs of these five antibiotics were no lower than 68.8% with mEs of 6.3–31.2%. Note, these plates were incubated for the same time span as the CCS AST assays (20 h) to avoid differences in MIC resulting from different incubation times^38^. These outcomes thus demonstrate the accuracy of the new method, not only for standard bacterial strains but also for isolates.

The MICs obtained by the CCS AST assay are almost identical to those obtained by standard BMD assays for five different types of antibiotics against isolates. Notably, given that the readout is effectively an operator-independent method (i.e., automated reading bypassing operator error^8^), it is not surprising that the CCS AST assay is more precise than the standard manual BMD^35^ (Table 2). In 72 percent of the counterpart assays, the MICs obtained with the CCS AST are higher than that obtained with the BMD AST, thus demonstrating that the automated method is more sensitive and thus more reliable. Moreover, versus endpoint measurements of BMD assays, the dynamic sensorgram obtained by the CCS offers more detailed information on the antibiotic activity at different growth stages^37^. For example, the CCS can show that the presence of ampicillin at a concentration below the MIC will stimulate *E. coli* growth. Notably, the EAs and mEs of these five antibiotics against *E. coli* are visibly different from *V. parahaemolyticus*, suggesting that the accuracy of the CCS AST assay also depends on the differences in the testing systems.

Versus automated optical methods, the CCS AST method has a few attractive features. For example, it is unnecessary to remove optical interference substrates from the liquid testing broth because they do not affect the CCS measurements^28^; this leads to a simpler operation. In addition, costs are much lower than optical systems. Electrochemical devices are cheaper than optical instruments because they do not require complex optical-electrical conversion^18^. The CCS system itself has an approximate price of $9000 (USD), which is much cheaper than that of VITEK and Phoenix. By contrast, the cost of assay consumables is rather low. Apart from broths and disposable tubes, neither chemical nor biotic auxiliary materials are needed for the AST assay. The cost of a single assay is no more than $1. Moreover, online monitoring to only detect the time point of transition from lag to exponential phase can save many hours^9^. High temporal resolution is important for accurate assays. The temporal resolution of common optical methods is around 10 min^9,38^. By employing Fourier transform reflectometric interference spectroscopy, the temporal resolution can reach 1 min^37^. In contrast, CCS collects a conductivity signal every 30 s to generate growth curves. When required, the temporal resolution can be set higher.

The CCS AST assay requires manual preparation of testing samples and incubation/automated readout. The first step is the same as the BMD AST assay. Operator error is possible because of the requirements for manual processes^8^. This might be the major contribution to the small variation of MICs. However, the preparation of inocula can be easily automated in a commercial version as described^15,35^. In addition, commercial model target antibiotics at a desired amount can be loaded into sample tubes beforehand with mass customization; thus, there are only two fully automated steps in the mature CCS AST assay.

In principle, the proposed CCS AST method successfully addresses issues facing automated optical methods. It does not suffer from interference from complex substrates in the test samples or from adherent cells in proliferation. We believe this enables it wider application fields. In some cases where automated optical methods are inadequate^38,42^ (e.g., blood culture systems, presence of microplastics, nanomaterials and silts in testing system, adherent bacteria), it can work well expectedly.

This study provides proof-of-concept of the phenotypic AST method based on the multichannel CCS. In the presence of antibiotics, dynamic processes of bacterial growth are monitored on-line in a non-destructive manner to generate growth curves. Thus, the MICs of antibiotics against target microorganisms are directly obtained. The total EAs between the CCS AST assays and the reference BMD AST assays are 68.8–92.3%; this demonstrates the accuracy of the new method for standard bacterial strains and isolates. This approach is superior to the BMD AST method in terms of simplicity, sensitivity and user-friendliness. The sensor itself is affordable. Moreover, the cost for applications is low because it does not involve expensive instruments or auxiliary chemicals. The proposed method provides an automated way to perform AST assays beyond situations where optical methods can be used. It is also a promising high-throughput tool. More validation experiments are planned, e.g., assays in the presence of blood, nanomaterials, silts or micro-plastics. We expect that these experiments will help clinical laboratories develop a versatile platform for rapid MIC determination of diverse types of microorganisms including adherent species.

## Supplementary information

Supplementary information.
