# Supplementary material for: Affordable automated phenotypic antibiotic susceptibility testing method based on a contactless conductometric sensor
Source: Sci Rep. 2020 Dec 4;10:21216. doi: 10.1038/s41598-020-77938-7 (PMC7718250; doi:10.1038/s41598-020-77938-7)
Supplement: Supplementary file 1 — Supplementary information. [file 41598_2020_77938_MOESM1_ESM.pdf]

Affordable Automated Phenotypic Antibiotic Susceptibility Testing Method Based on a Contactless Conductometric Sensor

Xuzhi Zhang[1,2], Xiaoyu Jiang[1], Qianqian Yang[1], Yong Xu[1], Xiaochun Wang[1], Jinping Wang[3]*, Xiaobo Sun[3], Guosi Xie[1], Yan Zhang[1], Jun Zhao[1], Keming Qu[1,2]*

[1] Yellow Sea Fisheries Research Institute, Chinese Academy of Fishery Sciences, Qingdao 266071, China

[2] Laboratory for Marine Fisheries Science and Food Production Processes, pilot National Laboratory for Marine Science and Technology (Qingdao), Qingdao 266071, China

[3] College of Chemical and Pharmaceutical Sciences, Qingdao Agricultural University, Qingdao 266109, China

Characterization of the 8-channel contactless conductometric sensor (CCS)

The properties of the 8-channel CCS were characterized using 0.12 M and 0.05 M KCl solutions at 25 ℃ with the same methods as we used previously[1]. As shown in **Fig. S1**, there are no visible apparent conductivity changes at all the response curves over a period of 20 hours. The variation from background interference is no more than 0.12%. These results demonstrate not only the uniformity of temperature in the chamber of the sensor, but also the robustness and reproducibility of the capacitively-coupled contactless conductivity detector system. The faint difference of apparent conductivity responses of the samples at the concentration result from minor variations in the geometry size of the tubes and their coupling to the electrodes[2]. Because that the critical factor is to monitor conductivity changes during the process of bacterial growth rather than determination of the absolute value, these minor variations does not interfere with the measurement by using an appropriate algorithm[1].

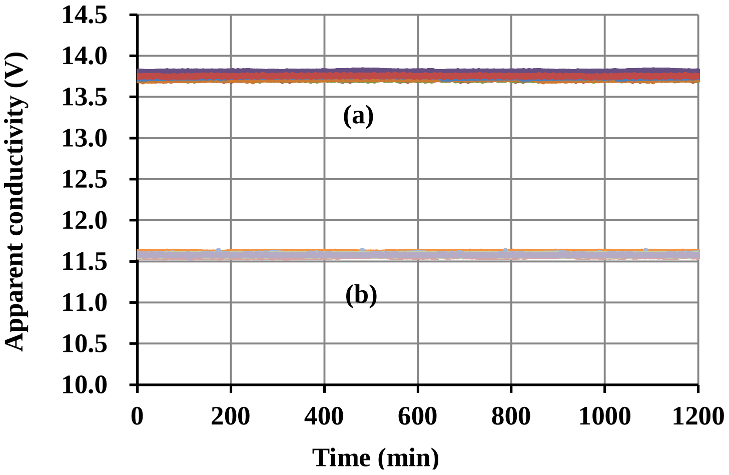

Figure S1 Typical apparent conductivity responses of 0.12 M (a) and 0.05 M KCl solution (b) recorded with the 8-channel CCS for 20 h at 25±1 ℃. A bath of 8 tubes was monitored simultaneously. In each of the tube 1.8 mL KCl solution was loaded. The apparent conductivity values were recorded after a 2 min incubation to balance the temperature inside and outside of the tubes. Operation parameter: excitation frequency of 1.0 MHz; excitation amplitude of 16 V; recording rate of 0.5 min.

(A： *E.coli*)

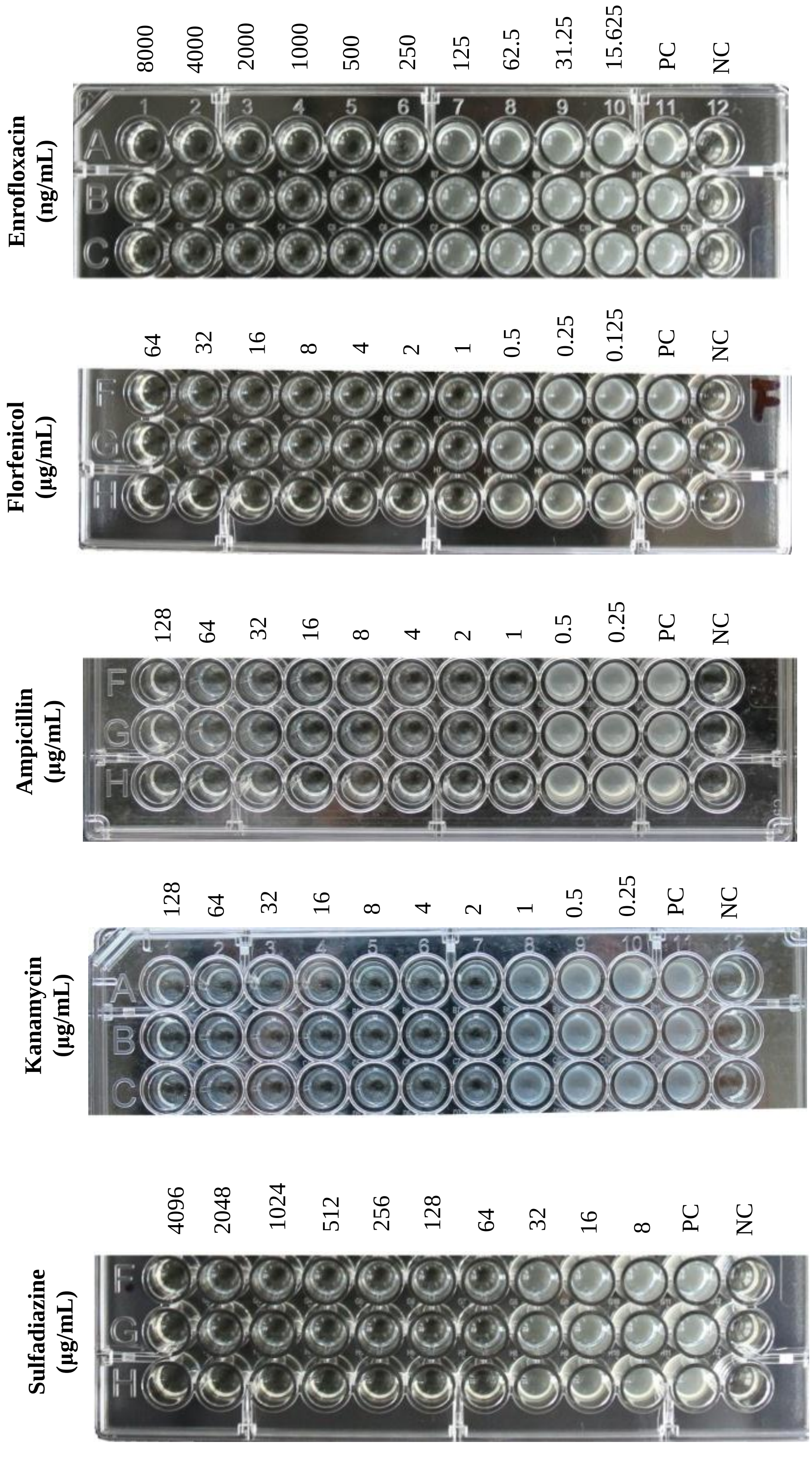
8000 4000 2000 1000 500 250 125 62.5 31.25 15.625 PC NC
Enrofloxacin (ng/mL)
64 32 16 8 4 2 1 0.5 0.25 0.125 PC NC
Florfenicol (µg/mL)
128 64 32 16 8 4 2 1 0.5 0.25 PC NC
Ampicillin (µg/mL)
128 64 32 16 8 4 2 1 0.5 0.25 PC NC
Kanamycin (µg/mL)
4096 2048 1024 512 256 128 64 32 16 8 PC NC
Sulfadiazine (µg/mL)

(B：*V. parahaemolyticus*)

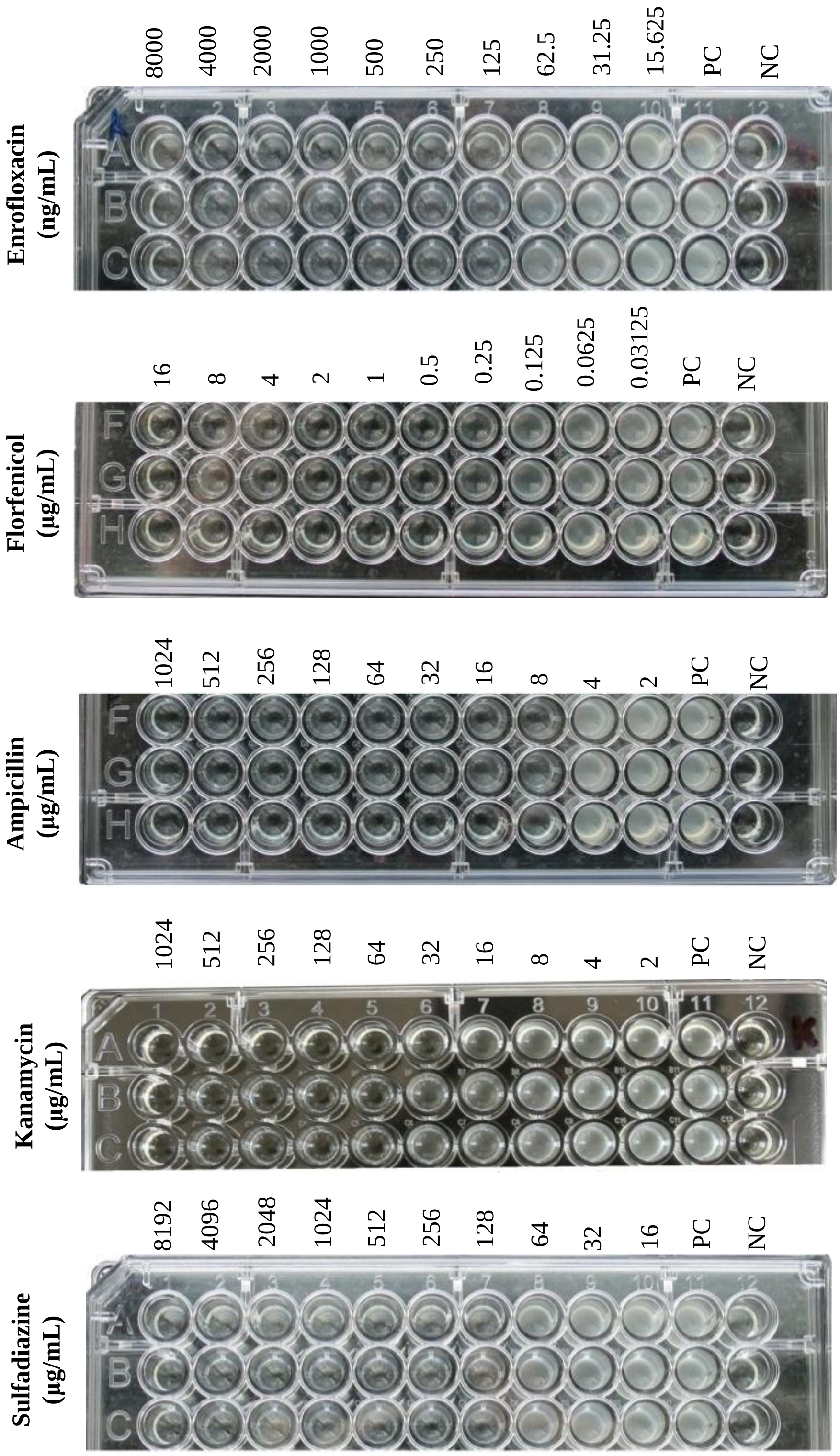

Figure S2 Results of BMD AST assay for *E. coli* (A) and *V. parahaemolyticus* (B) in 96-well plate. The final bacterial inoculum in the measurement was approximately 5×10^5 CFU/mL. In each well 100 μL LB or 2216E was used as nutrient broth for *E. coli* and *V. parahaemolyticus*, respectively. Plates were incubated at 37 ℃ for *E. coli* and at 28 ℃ for *V. parahaemolyticus*, for 20 h.

1. Zhang, X., *et al*. Online monitoring of bacterial growth with electrical sensor. *Anal. Chem*. **90**, 6006-6011 (2018).
2. Kubáň, P. & Hauser, P.C. Fundamental aspects of contactless conductivity detection for capillary electrophoresis. Part I: Frequency behavior and cell geometry. *Electrophoresis* **25**, 3387-3397 (2004).
